# Female-Driven Multiple Concurrent Sexual Partnership Systems in a Rural Part of a Southern Tanzanian Province

**DOI:** 10.1371/journal.pone.0145297

**Published:** 2015-12-18

**Authors:** Abela Mpobela Agnarson, Susanne Strömdahl, Francis Levira, Honorati Masanja, Anna Ekéus Thorson

**Affiliations:** 1 Department of Public Health Sciences, Karolinska Institutet, Stockholm, Sweden; 2 Ifakara Health Institute, Dar es Salaam, Tanzania; University of Stirling, UNITED KINGDOM

## Abstract

**Background:**

Multiple concurrent sexual relationships are one of the major challenges to HIV prevention in Tanzania. This study aims to explore sexual behaviour patterns including the practice of multiple concurrent sexual partnerships in a rural Tanzanian setting.

**Methods:**

This qualitative study used focus group discussions and in-depth interviews with men and women from the community as well as ethnographic participant observations. The data was collected during 16 months of fieldwork in 2007, 2008, and 2009. The focus group discussions and in-depth interviews were transcribed verbatim and translated into English. The data was analysed through the process of latent content analysis. An open coding coding process was applied to create categories and assign themes.

**Findings:**

*Mafiga matatu* was an expression used in this society to describe women’s multiple concurrent sexual partners, usually three partners, which was described as a way to ensure social and financial security for their families as well as to achieve sexual pleasure. Adolescent initiation ceremonies initiated and conducted by grand mothers taught young women why and how to engage successfully in multiple concurrent sexual relationships. Some men expressed support for their female partners to behave according to *mafiga matatu*, while other men were hesitant around this behaviour. Our findings indicate that having multiple concurrent sexual partners is common and a normative behaviour in this setting. Economical factors and sexual pleasure were identified as drivers and viewed as legitimate reason for women to have multiple concurrent sexual partnerships.

**Conclusions:**

Structural changes improving women’s financial opportunities and increasing gender equality will be important to enable women to not depend on multiple concurrent sexual partnerships for financial security. Future research should explore how normative sexual behaviour changes as these structural changes take place.

## Introduction

Tanzania has an overall HIV prevalence of 6% among the adult population (18–49 years old), and heterosexual transmission is the main mode of transmission [1]. Even though comprehensive HIV prevention interventions are now widely available, reducing the epidemic remains a challenging task. In a survey conducted in 2010, 29% and 16% of married or cohabitating men and women, respectively, in Tanzania reported having had extramarital sex in the previous 12 months [2].

Multiple concurrent sexual relationships are one of the major challenges to HIV prevention, and have been suggested to increase HIV transmission rates by creating sexual networks and by facilitating transmission chains during the acute stage of HIV infection [3, 4]. The concept of multiple concurrent sexual partnerships is defined as “overlapping sexual partnerships in which sexual intercourse with one partner occurs between two acts of intercourse with another partner” [5]. It has been reported that women from the poorest households are more likely to engage in multiple concurrent partnerships compared to women from the richest households in Sub-Saharan Africa (SSA) [6]. Further, a systematic review of 68 epidemiological studies from 1986 to 2006 on HIV infection rates found that women who reported three or more sex partners had three times the likelihood of acquiring HIV compared to women with up to two sex partners emphasizing the importance of number of sex partners in driving the epidemic [7].

In matrilineal descent groups in Tanzania, the woman’s maternal clan have been described to hold the greatest responsibility for her offspring, including that the maternal brother and uncle are responsible for her children [8–11]. The practice of *Unyago* initiation rites for girls (excluding female genital mutilation) and *Jando* initiations rites for boys have been described in anthropological data from the 1970’s among the matrilineal descent groups of the Zaramo, Ndengereko, Makonde, Hehe, Luguru, Kaguru, and Matumbi tribes [8–10, 12, 13]. *Unyago* is a system of informal education offered to girls, often conducted directly after the first menstrual event, by an older woman called *Kungwi*. Women who have passed *Unyago* are invited to participate in the ceremony. The lessons taught are regarding life skills including sexual behaviour and how to conduct relationships with male partners [12, 14–16]. This ceremony ensures the transfer of knowledge and practices from generation to generation [15, 16]. After passing the ceremony the adolescent girl is regarded as an adult [17–19]. Previous research underscore that initiation ceremonies are important in the process of socializing the child into specific social gender roles and thus hold the key to a proper and expected social life [20, 21]. Because of the sexual content in the teaching during *Unyago*, some scholars have argued that it contributes to sexual risk behaviour for HIV and other sexually transmitted infections (STI) among adolescents [22, 23].

Grey literature on sexual risk behaviour for HIV/STIs has previously reported on the use of the Swahili term, *mafiga matatu*, which means three stones in English and refers to an image of a three-stone fire with a cooking pot, in which all three stones are needed to balance the cooking pot. The term has been mentioned in connection to women who have multiple concurrent sexual partners. However, no definition for this term has been provided, neither has it been further described [14, 15, 24].

Structural factors such as poverty and gender inequality has previously been reported to motivate multiple concurrent sexual partnerships [25]. Largely missing from the discussion on multiple concurrent sexual partnerships is how the lessons and experiences on sexuality at a young age, by traditions such as unyago, play out in adult life [26]. In addition, HIV prevention programmes aiming to reduce multiple concurrent sexual partnerships needs to be informed by anthropological and qualitative studies [27].

This study aims to explore sexual behaviour patterns including the practice of multiple concurrent sexual partners and the term *mafiga matatu* in a rural Tanzanian setting.

## Methods

### Study setting

Rufiji district lays on a river delta in the Pwani region 180 kilometres south of Dar es Salaam in Tanzania. The district has a population of about 200,000 people distributed over 94 villages. The population is predominantly Muslim with a few Christians who are mainly immigrant labourers from other parts of Tanzania. The main ethnic group in the district is the matrilineal descent Ndengereko, and other groups include the matrilineal Zaramo, Matumbi, Ngindo, Pogoro, and Makonde [8, 9]. Swahili is the lingua franca throughout the district. Most of the population is self-employed in agriculture, fishing, or in small businesses alongside the district’s main transportation route; a trunk highway from Dar es Salaam in the north to Lindi in the south.

### Data collection

This qualitative study was conducted in 2007, 2008, and 2009 during a total of 16 months of fieldwork in 32 villages, largely by the first author who is fluent in Swahili. This study was a part of a collaboration between Rufiji Demographic Surveillance Site (DSS) and Karolinska Institutet that aimed to contribute to improve HIV care and interventions for reduction of sexual risk behaviour for HIV. Prior to each data collection phase, the first author visited all of the study sites to introduce the study and to make arrangements for interviews and discussions.

In depth interviews (IDIs) were conducted to gain a holistic understanding of the interviewee’s sexual relationships and opinion on multiple concurrent sexual partnerships [28]. Focus group discussions (FGDs) were performed in order to capture data based on group interactions in discussions on sexuality and societal norms [29, 30].

A purposeful sampling design was adopted to reach different groups in the societies regarding gender, age, socioeconomic status and important positions within the communities such as religious leaders and teachers. Local leaders and colleagues from the Rufiji DSS assisted in identifying key informants for IDIs and FGDs. The District Medical Officer assisted in recruiting healthcare worker to interview.

Four research assistants (three females and one male representing a diversity in age) were trained in interviewing technique and observation methodologies with the aim to achieve confidential culture-sensitive interviews on a private topic such as sexual behaviour [31, 32]. Interview guides in Kiswahili were used to facilitate the FGDs and IDIs that explored sexuality and sexual practices and the socio-economic and cultural contexts of sexual behaviour and livelihood. One research assistant and the first author assumed the role of the moderators (facilitators), and the other took the role of recorder (note taker) or observer. The most sensitive topics were deferred until later in the interview to give the interviewee sufficient time to become comfortable with the interview process and the questions being asked. A combined total of 91 individuals participated in 11 FGDs (men and women in the community) and 21 IDIs (including social workers, traditional healers, the initiation rites instructor (*Kungwi*) and her assistant *(fundi)*, religious leaders, and men and women in the community) (Table 1). FGDs were held separately for males and females to enhance spontaneous discussions. Uniformity of FGD participants in terms of age, socio-economic status, and educational background was taken into account. The number of participants in each group ranged from six to eight people, and the interviews were digitally recorded. All participants were informed about the objectives and procedures of the study.

**Table 1:** Data collection approach, time, and participant characteristics.

**INTERVIEWS**	
*2007*, *FIVE MONTHS*	
3 FGDs	21 men and women from the community (15–49 years of age)
4 IDIs	1 religious leader, 1 community leader, 1 social worker, and 1 political leader
*2008*, *NINE MONTHS*	
4 FGDs	25 men and women from the community (22–50 years of age)
9 IDIs	2 religious leaders, 1 teacher, 2 social workers, 1 political leader, 1 healthcare official, 1 traditional healer, and 1 initiation rites expert
*2009*, *ONE MONTH*	
4 FGDs	24 men and women in the community (22–47 years of age)
8 IDIs	4 men and 3 women from the community (30–48 years of age) and 1 Initiation rites expert (Kungwi)
**ETHNOGRAPHIC PARTICIPANT OBSERVATIONS, 2007–2009**	
*INFORMAL COVERSATIONS*	
Men and women from the community, local and religious leaders, truck and bus drivers, travellers, migrant workers, and bar and hostel owners	
*OBSERVATION*	
Seasonal activities such as farming, initiation rites, traditional healing, religious festivals, and extramarital activity negotiation and income practice. Marriage and divorce negotiations.	
Income-generating sexual activity in local bars and during *Ngoma* and *Rusha roho* parties	
"Supu ya mawe"-"soup of stone" brewing and manufacturing activity (liquor brewed from cashew apple). The main drink at *Rusha roho* parties.	
*PARTICIPANT OBSERVATION*	
Traditional healing activities	
Religious gatherings & festivals	
*Ngoma* (night festivities in connection to *Unyago*)	
Kitchen parties (Bride rites of passage).	
Wedding parties	
Discos	
"*Rusha Roho*" = "Make your heart happy"—all night parties	

Developing an accurate and complete insight into culturally embedded issues requires methods, such as participant observation, that are able to show the extent and diversity of social constructs [33]. Participant observations (PO) and informal conversations were conducted from a cultural relativism standpoint by the first author and one research assistant. This allowed for a more relevant understanding of the context in which the events or conversations took place [32]. The aim of the participant observations was to gain further understanding of the *‘mafiga matatu’* and female multiple concurrent sexual relationships. The research team participated and observed diverse social activities, including initiation and traditional healer ceremonies, kitchen parties, wedding parties, and “from dusk to dawn parties” (Ngoma and rusha roho). Spontaneous conversations and discussions were conducted in local bars, hair salons, buses, barbershops, and while attending discos or parties, which led to invitations to activities within the society. Being newcomers to a small village meant that there was curiosity to get to know the newcomers which opened up doors to many social venues. The first author spent afternoons with men in the village carpenting shops and sport clubs and spent evenings with female food vendors alongside the main road. The research team spent substantial amounts of time with traditional healers (*Kungwi*) and local leaders in their working environments. Furthermore, the first author was invited into people’s homes and took part in the family’s daily chores such as farming during harvest season. The notes were summarized after the *who*, *what*, *when*, *why*, and *how* system that is often used in the field of journalism [34].

### Data analysis

Data from the IDIs and FGDs were transcribed verbatim and translated into English, and the data were analysed through the process of latent content analysis. The material was read repetitively followed by an iterative process of open coding, creating categories, and assigning themes by two independent researchers (AM, SS) using Open Code software [35]. Specifically, we used the constant comparative method to identify the latent pattern in multiple participants’ perspectives. These perspectives were categorized primarily by the words that the participants had used. At the same time, we reviewed line, sentence, and paragraph segments of the transcribed interviews. Each code was continuously compared to all other codes to identify connections, dissimilarities, and common patterns. Discrepancies between codings were resolved through discussions between the authors and the research assistants who were involved in the data collection.

The ethnographic material, consisting of fieldwork journals, notes, jottings, pictures, and drawings, were indexed using mind-mapping techniques in the FreeMind version 05 software package. The analysis process involved reflecting on words, notable sentences, phrases and expressions, and descriptions of context and events. Finally, the coded field notes were reviewed to assign appropriate codes to the concepts that had been suggested by the IDI and FGD data. Triangulation of these methods was used to capture comprehensive qualitative data on a sensitive and private topic such as sexual behaviour.

### Ethics statement

The Medical Research Coordinating Committee and the Tanzania Commission for Science and Technology (COSTECH) (NIMR/HQ/R.8a/VolIX/609) approved the study and consent procedures. Study permission was also sought and approved by the District Community Officer and village leaders prior to engaging in fieldwork. Further meetings were held with village leaders during each study period to inform them about the process and up-coming steps of the study. Informed consent was explained to each respondent and written (thumb print) consent was granted prior to conducting any formal interview. Oral consent was given for participant observations. Participants were free to refuse to participate and to withdraw at anytime without providing a reason.

To ensure anonymity, no names or identity information of participants or villages are included in the final manuscript.

## Findings

The findings of this study are presented according to the themes and categories that emerged from the data analysis. The initial coding produced separate categories, and examination of the patterns and relationships between these categories resulted in sub-themes underlying female multiple concurrent sexual partnerships. A detailed description of the sub-themes together with quotations from the research participants and descriptions from ethnographic participant observations are presented. In some quotations the Kiswahili phrases are kept to retain the research participants’ voices and to contextualize the findings. This is especially the case where a precise translation to English has been difficult to achieve. Specific details have been modified to protect the confidentiality of the research participants, and spaced dots indicate where lines have been omitted. The participants’ gender, source of data (IDI, FGD, PO) and age group (young 18–25, middle-aged 25–40 and older ≥40 years of age) is presented with quotes or PO data. All participants were either married, co-habitating or in a sexual relationship.

### A social insurance system based on sexual relations

#### Mafiga matatu as described in the study setting

*Mafiga matatu* is an expression used in this society to describe female’s multiple concurrent sexual partnerships, and the term was used in everyday conversations. The norm around female multiple concurrent sexual partnerships was found to include having three male sexual partners (three stones). It was described that a women needs three male partners in order to be balanced in life and content i.e. the optimal partner situation. During the *Unyago* ceremony young women and girls were taught about the structure around *mafiga matatu* including why you should have and how to conduct multiple concurrent sexual partnerships as a woman. Female participants explained that to not engage in *mafiga matatu* i.e. having three concurrent sexual male partners, means that one is prone to suffer from hunger and emotional starvation.

PO revealed that the argument made for experiencing hunger and emotional starvation without concurrent sexual relations, was grounded in the teachings from *Unyago*. A song commonly sung during these initiation ceremonies says:

*“Keep the balance in your house*, *children should not starve [watoto wasife njaa]*, *keep one foot in the house and one foot outside [mguu mmoja mbirini*, *na mguu mmoja nje]*.*”*

These lines confirm that the argument for starvation is grounded in the teachings of the initiation rites and is used to motivate engagement in *mafiga matatu* as insurance against starvation. The lovers or backups in this case should be treated with care so that the “*reserve will not decay* [akiba haiozi]” according to instructions given to a young woman by the older women at an initiation ceremony.

#### An example of the practice of mafiga matatu

**Fig 1:**
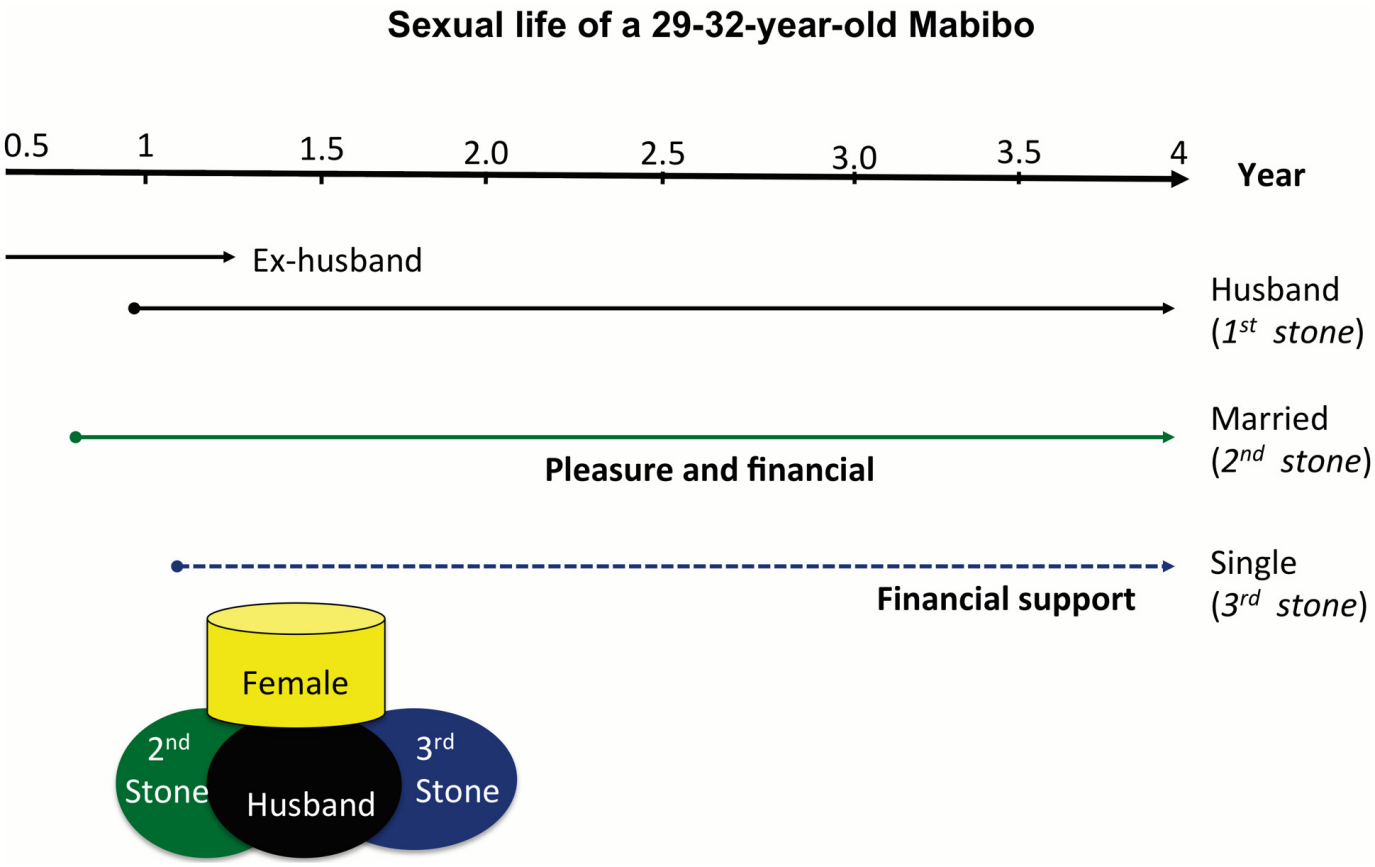
Description of the *mafiga matatu* system of a married woman, from 29 to 32 years of age. The figure illustrates the structure of *mafiga matatu*, a wife balancing on top of the husband or steady partner and two lovers.

Fig 1 illustrates the *mafiga matatu* with an example of a period in a woman’s life when she was 29 to 32 years of age. She had a steady employment with a monthly salary of around 50,000 sh/TSZ, equivalent to 31 US$ (the average salary for a government employee is about 125 US$ per month). The woman was married to a farmer whom she suspected of having a mistress in a neighbouring village. She became involved with her *“2* ^*nd*^ *ston*e”, a married local man who held a steady job in a local organization, before marrying her current husband. Her *“2* ^*nd*^ *ston*e”, provided her with sexual pleasure and financial support. Her *“3* ^*rd*^ *stone”* was a self-employed man, and the relationship started after her current marriage. She referred to him as mvumba–‘fish smell,’ local slang for a man with money or Kabwela (single man). We were not informed about the length of the relationship, but it was established that he provided her with financial support. The woman describes her relationship with the *“3* ^*rd*^ *stone” below;*

*“He brings me big fish* [money], *maintenance*, *other times he will help my children*. *When I have nowhere to find help or support*. *He smells fish* [money], *(*…*) he knows how to take care of my middle path-my vagina* [njia yangu ya katikati].”

#### Older women’s role in unyago

Older women led the *Unyago* ceremony and explained that they were passing on the teachings they had been taught as children. During PO they explained that *Unyago* was a necessity for young women and girls in today’s society in order to become and be viewed as adults. Paternal and maternal grandmothers have the mandate to organize initiation rites. Female sexuality is described to young women by older women during *Unyago* initiation ceremonies, as pleasurable, profitable, and the foundation for biological continuity. Sexual pleasure for both men and women was emphasized and young women were instructed on how to perform sex and the importance of pleasure for the woman (and man) during sex. This conceptualization was referenced in practice and by demonstration during the *Unyago* session. This could go as follows: the *fundi* (the technical assistant to the *kungwi*) adopted the role of *mwanamke nyonga* (a female expert in coital conduct) to instruct the *mwali* (the young woman being initiated) on coital technique. During the session, the *kungwi* ensured that the initiate, young woman, repeated the *fundis’* meticulous pelvic movements while giving remarks to the *mwali* such as, *“Sex is an issue of pleasure*, *it gives you a child*, *it gives you pleasure*, *it is a path to assets*.*”*

#### Women seeking sexual pleasure through mafiga matatu

Female and male informants both viewed sexual pleasure as a legitimate reason to seek extramarital relations, exposing that it is partly the lack of sexual pleasure in their primary relationships that contributes to extramarital relations. For some women, some extramarital relations were conducted for pure pursuit of sexual pleasure, as articulated by a middle-aged woman in an IDI:

*“I can’t depend on men (…) to settle my problems*. *I will work hard on my own (…) I secure a lover only to relax and this thing [sex for pleasure] is normal*. *I can’t depend on men (…) the backbone of my life is to depend on myself*.*”*

A common theme among the participants in the FGDs and IDIs was that women looked for lovers because of sexual dissatisfaction in polygamous unions. They viewed it as their right to seek extramarital sex partners to satisfy their sexual needs and gain sexual health, describing themselves as their own agents of sexual happiness. A young woman describes her situation in an FGD:

*“I am in a polygamous marriage*, *(…) we are four wives*, *my husband makes a round of seven days (…)*, *and for how long will I be without sex*? *I have to wait twenty-one days*. *I’m still fresh*, *can I tolerate that [not having sex]*?*”*

Other women described their spouse’s intolerance to their sexual needs as motivation to seek sexual pleasure and to find a lover outside of the marriage. Thereby exposing multiple reasons for women to have multiple concurrent sexual partners. The female participants described the importance of sexual satisfaction to them and the need to search for sexual partners that could satisfy them. Once again, describing themselves as active agents in searching for and finding sexual satisfaction as stated by a middle-aged women in a FGD.

*“Our men don’t appreciate when the wife wants to enjoy sex*, *they will fight with you*. *As a woman*, *I’m suffering at home*, *outside the marriage I find comfort and pleasure*.*”*

The female participants expressed agency regarding their own sex life and well-being. In addition, women supported and empowered each other to do so.

#### Women seeking financial support through mafiga matatu

Participants explained that it was a necessity to engage in *mafiga matatu* and have multiple concurrent sexual partners for emotional and financial reasons. A young woman put forward an argument in agreement with others in an FGD:

*“In Christianity*, *it is not acceptable to have other men once the person is married (…) before a person decides not to take on another man*, *that person needs first to realize that by not having another man*, *they will starve physically and emotionally [wao watakufa njaa ya kimwili na kihisia]*.*”*

Female informants explained the need to plan and strategize extramarital activities in a way that will generate good and sustainable support. Women often used different metaphoric descriptions to explain the transaction management involved in multiple concurrent sexual partnerships, *mafiga matatu*. One middle-aged woman in a FGD compared the financial management in the *mafiga matatu* system with a family planning program:

*“You know in this place we have this program of rearing children through the scheme of the green star [nyota ya kijani*, *a family planning campaign in the 1990s]*. *We say*: *I breed through planning; I nurture with pride*. *In respect to our mafiga matatu*, *we stick to intercourse with a plan [ngono yenye mpango]*.*”*

Women that constantly changed lovers were not respected by other women in the community because they did not follow the *mafiga matatu* way. In one FGD session, one middle-aged woman described the perceived deviant behaviour of changing lovers as *“she transports one trip gravel*, *the next trip is sand [anabeba trip kokoto*, *trip mchanga]*.*”* The metaphoric use of gravel and sand means that there was little financial and no social gain in having several short-term sexual relationships and that no status could be achieved by brief sexual relationships.

The *ngono yenye mpango* instructions given during *Unyago* as according to PO data, declare that a woman should not financially drain the lover, so financial requests should be made only occasionally and only when in need. The extramarital male partners provide financial security for times when the woman and her family might be in need of financial help in the future. An older woman in a FGD explained this through the metaphor below:

*“A skilled farmer does not cultivate the same crop on the same land each season [Mkulima mwenye ujuzi*, *hakulima mazao juu ya ardhi moja kwa kila msimu] (….) it does not give a good harvest because that soil lacks nutrients for a good harvest*, *(*….*) during one season I cultivate peas (*….*) then the next season we allow the soil to regenerate*, *(*….*) as we relocate the crop to a different area (*….*) it's the same thing with establishing assistance from lovers*.*”*

Many women viewed the practice of *mafiga matatu* as an ideology of sexuality that sees women as agents of their own sexuality and economic fulfilment. A middle-aged female food vendor explained that she justified her engagement in lovers because of economic needs as well as an effort to secure sexual pleasure and temporary comfort:

*“I would like to have one man (…) sometimes the man whom you are living with will not help (…) I do follow my culture (*…*) for me having secret lovers is all about hunger (…) the chance to relax my soul and have a good time*, *but there is no true love in that”*

#### Married men promoting mafiga matatu

Research participants explained that the man was the head of the household. However, it was widely acknowledged that the man’s spouse would not attain stability in life if she were to depend financially and emotionally only on her husband or primary male partner. According to some male participants, women’s extramarital relations were considered an indication of commitment to the primary male partner and proved that their woman had good survival skills. An older male participant, who at time of the interview was involved in two other relationships outside his marriage, explained the purpose of *mafiga matatu*:

*“The man has the authority*, *and a wife has her place in terms of mafiga matatu*. *If she only relies on me she might not be stable*, *it is not possible for the woman to stay in that relationship*. *There will be times I will be out in the farm*, *(*….*) preparing charcoal or sick*. *She will not have any help if she is not engaged in in mafiga matatu (…)”*

Men also declared in PO data that extramarital activities were not a secret per se in the community, even though it was not explicitly talked about. Both men and women described a certain responsibility in maintaining discretion in regards to extramarital relations. Indiscretion was deemed as a woman’s inability to behave and practice according to her training at *Unyago*. A middle-aged male participant in an IDI described how a woman’s indiscretion about her extramarital activity, not the actual extramarital sexual relation, was the basis for discord in marriages:

*“Many marriages experience difficulties because of the improper practice of mafiga matatu*. *It all depends on how competent the woman is*…*”*

#### Married men hesitating around mafiga matatu

A diversity of opinion around *mafiga matatu* among male informants was found. A few men reported that they did not prefer their wife to practice *mafiga matatu*. For example, one man decorated the bedroom with HIV information posters as a reminder to his wife on the importance of fidelity. Another middle-aged man in an IDI emphasized that by being true to one’s wife and working together, mafiga matatu could be avoided in their marriage.

***“*** *When I’m working*, *she is on the farm cultivating bananas*, *harvesting and preparing cassava (*…*) selling chicken there at home (*…*) when I get back from work (*….*) we sit down and count together what we got that day (*….*) she keeps some money in savings (*…*) some she takes to buy clothes (*….*) we all spend together (*….*) in case I’m sick she takes part of the crops from the farm to sell and cover the expenses*.*”*

One young man reported in an IDI, who also had lovers outside of marriage, reported making financial transactions to his wife to keep her from having concurrent extramarital sex partners.

*“I have a Kisado [small house*, *lover]*, *to the wife at home I leave her some money so as she doesn’t go and sleep with someone else*. *The aim is to make sure she has money so that she doesn’t develop that desire to get the money from someone else*. *I give twenty percent to the woman outside [the lover] and fifty percent to my wife at home because she is the mother of my children*.*”*

Various participants in the study (PO) explicitly disapproved of the practice but covertly either engaged in the practice or supported the practice.

#### The impact of parents, religious, and political will on mafiga matatu

Men and women agreed that the instituted practice of *mafiga matatu* has its origin in the teachings at *Unyago*. Respondents in the study believed that religious leaders, who were denouncing the traditional practices, lack the power to abolish the practice or to change behaviour patterns. The paternal and maternal grandmothers hold the mandate to organize initiation rites and disapproving of their wishes was disrespectful. One middle-aged male participant reported this concern in an FGD:

*“Religious people are strongly informing against the practice (mafiga matatu)*. *They run war against it*, *(…) they cannot eliminate it because these leaders are children of a woman (…) my daughter experienced her first menstruation; my mother wanted Unyago*. *I cannot tell my mother that I don’t want my child to go through Unyago (*….*) I tried*, *it is like disrespecting your mother (…) because she tells me; “I was initiated in those savage practices*, *and I gave birth to you*, *what wrong did that do you*?*” You cannot go against your mother (…) Imams and priests alike have a mother too*.*”*

There were individuals from various local authorities, especially men, who strongly campaigned for both mafiga matatu and Unyago as a way of preserving ancestral culture. A middle-aged local male leader explained:

*“Mafiga matatu as I know it*, *to me it is a good learning*. *Because the elders have the learning*, *and we are in this situation now [referring to HIV]*, *but we respect our culture*. *It is one of the reasons we encourage the elders to continue with the teaching today*. *I cannot tell a mother of a person not to initiate her child or teach her about mafiga matatu*, *she will not understand me*.*”*

## Discussion

An in-depth understanding of the social patterns that favour multiple concurrent sexual partnerships is crucial for the design and implementation of effective behavioural HIV prevention programs. The findings in this study have generated insight into a social custom that circumvents the context of HIV in rural settings in southern Tanzania. It provides a deeper exploration on metaphors and system around multiple concurrent sexual partnerships. The findings indicate that the social guidance that originates from the ceremonial adolescence initiation rites for girls, *Unyago*, supports and encourages women’s choices to have multiple concurrent sexual partners, and these relations are termed *mafiga matatu* in the society. The older women in this society hold power in that they are the one initiating and conducting the initiation ceremonies during which young women are skilled in the system of practicing *mafiga matatu*.

The simplicities and complexities underlying the practice of *mafiga matatu* were contradicting each other. Participants described the practice as a long-term investment, a separate system from sex for procreation that introduces financial stability in the family [36]. Other participants disapproved of the practice, but recognized that these transactional relationships are nourished through traditional forces, which are difficult to ignore.

In the context of this matrilineal descent society, the need of a social and financial insurance system and sexual satisfaction were described as important drivers of multiple concurrent sexual partners. Both female and male participants regarded sexual pleasure as a motivation to engage in extramarital relationships. This supports previous findings from a qualitative study reporting the difference between sexual relations outside the marriage for pleasure compare to sex within marriage for procreation [36].

Better educational opportunities for women and conditional cash payments for schooling programs have been shown to decrease HIV incidence and delay the onset of sexual activity in SSA countries by providing young women with the means to make choices that decrease their HIV risk [37]. A recent study reported that sexual education has had a valuable impact on knowledge of HIV/STIs and condom use, but had little impact on attitudes towards multiple concurrent sexual partners among unmarried adolescents in one region of Tanzania, indicating other motivations for multiple concurrent sexual partnerships [38].

Primary education is mandatory in Tanzania for children ages 7–15 and public school fees have been eliminated since 2001, contributing to better education opportunities for girls [39]. Structural factors such as gender inequality and limited economical opportunities for women plays an important role in upholding multiple concurrent partnerships. As women’s rights and opportunities are improved in this setting the need for a social and financial security system through *mafiga matatu* may diminish.

Stoebenau concludes in a multi-country SSA study that the constituent of transactions within multiple sexual partnership is connected to the processes of globalization consumerism [40]. In contradiction, the data reported here implies a tradition of multiple concurrent sexual partnerships. The *unyago* ceremonies described in participant observation data reported here are similar to literature published in the 1970’s on this topic, indicating similar initiations rites over time [8,9,11,12].

The data presented here reflects upon one rural part of a southern Tanzanian province during the time data was collected. Several participants describe *mafiga matatu* as according to tradition or as according their culture. However, it is not possible to draw firm conclusions regarding cultural basis of the *mafiga matatu* practice based on these data. Sexual behaviour is dynamic and may change over time, therefore formative work prior to designing HIV prevention programs are key for successful development of the programmes and implementation thereof. A previous study from the Meru in Northern Tanzania, report that HIV prevention programs that does not resonate with local normative social behaviour around sexual practices such as multiple concurrent partnerships may be inefficient [41].

Further, a previous study from Mwanza in northern Tanzania reports that discourses around multiple concurrent sexual relationships are transmitted across generations, which may be used to design compelling HIV prevention programmes [26]. The *mafiga matatu* system has a strong foothold in this community, there may be a risk that prevention messages involving a decrease in number of sexual partners could be interpreted as disallowing this practice. One way forward is to value the initiation rites and at the same time modify its content in terms of including HIV prevention. During initiation ceremonies women across generations are under the same roof to talk about sexual relationships, this provides a possible venue to explore where messaging on HIV/STI prevention could be incorporated within the traditional agenda. However, educational interventions alone may not change the practice of having multiple concurrent sexual partnerships. In addition, the structural driver of limited economical opportunities for women in this setting needs to be addressed. Future research should explore how normative sexual behaviour changes as these structural changes take place.

## Conclusion

Having multiple concurrent sexual relations in rural part of southern Tanzania is a social behaviour that provides a financial and social security system for women and their families. This concept is called *‘mafiga matatu’*, which translates to ‘three stones’ and means that ‘you need three men to be stable’ i.e. three concurrent male sexual partners We found that the *mafiga matatu* system is taught to girls during initiation rites and through social interactions within the community. The findings indicate that economical factors and sexual pleasure were viewed as legitimate reason for women to have multiple concurrent sexual partnerships. Structural changes improving women’s financial opportunities and increasing gender equality will be important to enable women to not depend on multiple concurrent sexual partnerships for financial security.
